# Bit-Table Based Biclustering and Frequent Closed Itemset Mining in High-Dimensional Binary Data

**DOI:** 10.1155/2014/870406

**Published:** 2014-01-30

**Authors:** András Király, Attila Gyenesei, János Abonyi

**Affiliations:** ^1^Department of Process Engineering, University of Pannonia, Veszprém 8200, Hungary; ^2^Bioinformatics & Scientific Computing Core, Campus Science Support Facilities, Vienna Biocenter, 1030 Vienna, Austria

## Abstract

During the last decade various algorithms have been developed and proposed for discovering overlapping clusters in high-dimensional data. The two most prominent application fields in this research, proposed independently, are frequent itemset mining (developed for market basket data) and biclustering (applied to gene expression data analysis). The common limitation of both methodologies is the limited applicability for very large binary data sets. In this paper we propose a novel and efficient method to find both frequent closed itemsets and biclusters in high-dimensional binary data. The method is based on simple but very powerful matrix and vector multiplication approaches that ensure that all patterns can be discovered in a fast manner. The proposed algorithm has been implemented in the commonly used MATLAB environment and freely available for researchers.

## 1. Introduction

One of the most important research fields in data mining is mining interesting patterns (such as sequences, episodes, association rules, correlations, or clusters) in large data sets. Frequent itemset mining is one of the earliest such concepts originating from economic market basket analysis with the aim of understanding the behaviour of retail customers, or, in other words, finding frequent combinations and associations among items purchased together [1]. Market basket data can be considered as a matrix with transactions as rows and items as columns. If an item appears in a transaction it is denoted by 1 and otherwise by 0. The general goal of frequent itemset mining is to identify all itemsets that contain at least as many transactions as required, referred to as minimum support threshold. By definition, all subsets of a frequent itemset are frequent. Therefore, it is also important to provide a minimal representation of all frequent itemsets without losing their support information. Such itemsets are called frequent closed itemsets. An itemset is defined as closed if none of its immediate supersets has exactly the same support count as the itemset itself. For comprehensive reviews about the efficient frequent itemset mining algorithms, see [2, 3].

Independently of frequent itemset mining, biclustering, another important data mining concept, was proposed to complement and expand the capabilities of the standard clustering methods by allowing objects to belong to multiple or none of the resulting clusters purely based on their similarities. This property makes biclustering a powerful approach especially when it is applied to data with a large number of objects. During recent years, many biclustering algorithms have been developed especially for the analysis of gene expression data [4]. With biclustering, genes with similar expression profiles can be identified not only over the whole data set but also across subsets of experimental conditions by allowing genes to simultaneously belong to several expression patterns. For comprehensive reviews on biclustering, see [4–6].

**Figure 1:**
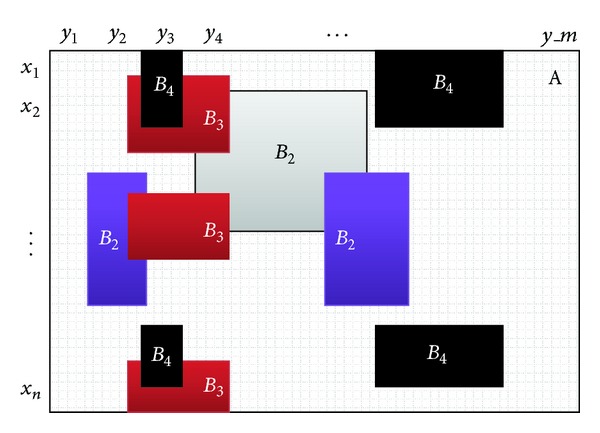
Illustrative representation of biclusters/frequent closed itemsets on binary data.

One of the most important properties of biclustering when applied to binary (0,1) data is that it provides the same results as frequent closed itemsets mining (Figure 1). Such biclusters, called *inclusion-maximal biclusters* (or *IMBs*), were introduced in [7] together with a mining algorithm, BiMAX, to discover all biclusters in a binary matrix that are not entirely contained by any other cluster. By default an *IMB* can contain any number of genes and samples. Once additional minimum support threshold is required for discovering clusters having at least as many genes as the provided minimum support threshold (i.e., minimum number of genes), BiMAX and all frequent closed itemset mining methods result in the same patterns.

In this paper we propose an efficient pattern mining method to find frequent closed itemsets/biclusters when applied to binary high-dimensional data. The method is based on simple but very powerful matrix and vector multiplication approaches that ensure that all patterns can be discovered in a fast manner. The proposed algorithm has been implemented in the commonly used MATLAB environment, rigorously tested on both synthetic and real data sets, and freely available for researchers (http://pr.mk.uni-pannon.hu/Research/bit-table-biclustering/).

## 2. Problem Formulation

**Figure 2:**
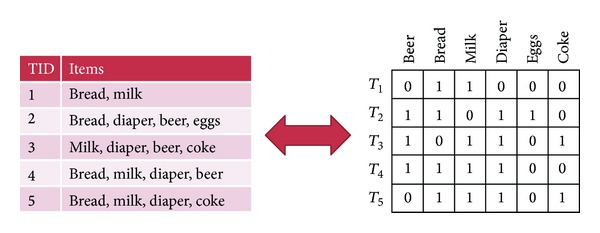
Bit-table representation of market basket data.

In this section we will show how both market basket data and gene expression data can be represented as bit-tables before providing a new mining method in the next section. In case of real gene expression data, it is a common practice of the field of biclustering to transform the original gene expression matrix into a binary one in such a way that gene expression values are transformed to 1 (expressed) or 0 (not expressed) using an expression cutoff (e.g., twofold change of the log2 expression values). Then the binarized data can be used as classic market basket data and defined as follows (Figure 2): let *T* = {*t*_1_,…, *t*_*n*_} be the set of transactions and let *I* = {*i*_1_,…, *i*_*m*_} be the set of items. The *Transaction Database* can be transformed into a binary matrix, **B** ^0^, where each row corresponds to a transaction and each column corresponds to an item (right side of Figure 2). Therefore, the bit-table contains 1 if the item is present in the current transaction and 0 otherwise [8].

**Pseudocode 1:**
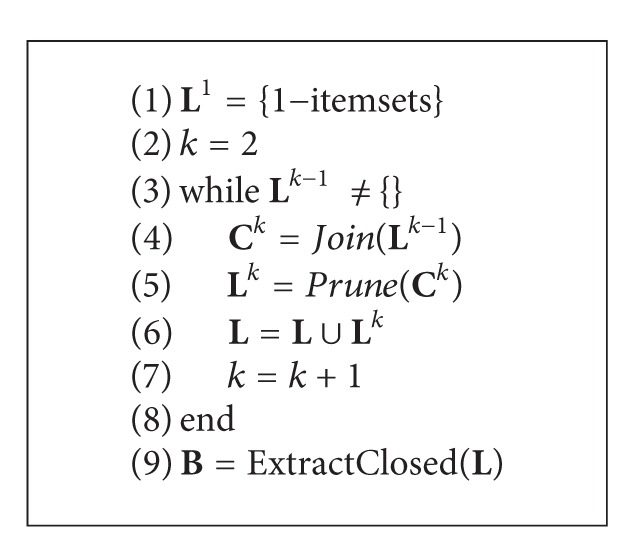
Pseudocode of the Apriori-like algorithm.

Using the above terminology, a transaction *t*_*i*_ is said to support an itemset *J* if it contains all items of *J*; that is, *J*⊆*t*_*i*_. The support of an itemset *J* is the number of transactions that support this itemset. Using *σ* for support count, the support of itemset *J* is *σ*(*J*) = |{*t*_*i*_ | *J*⊆*t*_*i*_, *t*_*i*_ ∈ *T*}|. An itemset is frequent if its support is greater than or equal to a user-specified threshold sup⁡(*J*) ≥ minsupp. An itemset *J* is called *k-itemset* if it contains *k* items from *I*; that is, |*J*| = *k*. An itemset *J* is a *frequent closed itemset* if it is frequent and there exists no proper superset *J*′⊃*J* such that sup⁡(*J*′) = sup⁡(*J*).

The problem of mining frequent itemsets was introduced by Agrawal et al. in [1] and the first efficient algorithm, called Apriori, was published by the same group in [9]. The name of the algorithm is based on the fact that the algorithm uses prior knowledge of the previously determined frequent itemsets to identify longer and longer frequent itemsets. Mannila et al. proposed the same technique independently in [10], and both works were combined in [11]. In many cases, frequent itemset mining approaches have good performance, but they may generate a huge number of substructures satisfying the user-specified threshold. It can be easily realized that if an itemset is frequent then all its subsets are frequent as well (for more details, see “downward closure property” in [9]). Although increasing the threshold might reduce the resulted itemsets and thus solve this problem, it would also remove interesting patterns with low frequency. To overcome this, the problem of mining frequent closed itemsets was introduced by Pasquier et al. in 1999 [12], where frequent itemsets which have no proper superitemset with the same support value (or frequency) are searched. The main benefit of this approach is that the set of closed frequent itemsets contains the complete information regarding its corresponding frequent itemsets. During the following few years, various algorithms were presented for mining frequent closed itemsets, including CLOSET [13], CHARM [14], FPclose [15], AFOPT [16], CLOSET+ [17], DBV-Miner [18], and STreeDC-Miner [19]. The main computational task of closed itemset mining is to check whether an itemset is a closed itemset. Different approaches have been proposed to address this issue. CHARM, for example, uses a hashing technique on its TID (transaction identifier) values, while AFOPT, FPclose, CLOSET, CLOSET+, or STreeDC-Miner maintains the identified detected itemsets in an FP-tree-like pattern-tree. Further reading about closed itemset mining can be found in [20].

The formulations above yield the close relationship between closed frequent itemsets and biclusters, since the goal of biclustering is to find biclusters *B*_*k*_ = (*I*_*k*_, *J*_*k*_), such that *I*_*k*_⊈*I*_*l*_, *J*_*k*_⊈*J*_*l*_. Therefore, while the size restriction for columns in a bicluster corresponds to the frequency condition of itemsets, the “maximality” of a bicluster corresponds to the closeness of an itemset. Thus, if itemsets that contain less than *min_rows* number of rows are filtered out, the set of all closed frequent itemsets will be equal to the set of all maximal biclusters.

## 3. Mining Frequent Closed Itemsets Using Bit-Table Operations

**Figure 3:**
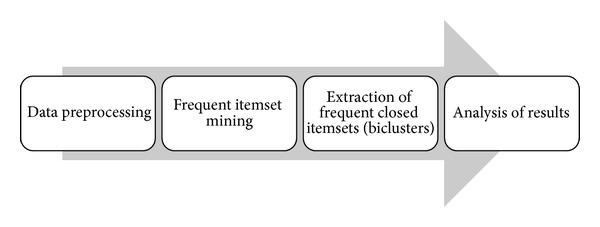
Schematic view of frequent closed itemset discovery.

In this section we introduce a novel frequent closed itemset mining algorithm and propose efficient implementation of the algorithm in the MATLAB environment. Note that the proposed method can also be applied to various biclustering application fields, such as gene expression data analysis, after a proper preprocessing (binarization) step. The schematic view of the proposed pipeline is shown in Figure 3.

### 3.1. The Proposed Mining Algorithm

The mining procedure is based on the Apriori principle. Apriori is an iterative algorithm that determines frequent itemsets level-wise in several steps (iterations). In any step *k*, the algorithm calculates all frequent *k*-itemsets based on the already generated (*k* − 1)-itemsets. Each step has two phases: candidate generation and frequency counting. In the first phase, the algorithm generates a set of candidate *k*-itemsets from the set of frequent (*k* − 1)-itemsets from the previous pass. This is carried out by joining frequent (*k* − 1)-itemsets together. Two frequent (*k* − 1)-itemsets are joinable if their lexicographically ordered first *k* − 2 items are the same and their last items are different. Before the algorithm enters the frequency counting phase, it discards every new candidate itemset having a subset that is infrequent (utilizing the downward closure property). In the frequency counting phase, the algorithm scans through the database and counts the support of the candidate *k*-itemsets. Finally, candidates with support not lower than the minimum support threshold are added into the set of frequent itemsets.

A simplified pseudocode of the Apriori algorithm is presented in Pseudocode 1, which is extended by extracting only the closed itemsets in line 9. While the *Join*() procedure generates candidate itemsets *C* ^*k*^, the *Prune*() method (in row 5) counts the support of all candidate itemsets and removes the infrequent ones.

The storage structure of the candidate itemsets is crucial to keep both memory usage and running time reasonable. In the literature, hash-tree [9, 11, 21] and prefix-tree [22, 23] storage structures have been shown to be efficient. The prefix-tree structure is more common, due to its efficiency and simplicity, but naive implementation could be still very space consuming.

Our procedure is based on a simple and easily implementable matrix representation of the frequent itemsets. The idea is to store the data and itemsets in vectors. Then, simple matrix and vector multiplication operations can be applied to calculate the supports of itemsets efficiently.

To indicate the iterative nature of our process, we define the input matrix (**A**_*m*×*n*_) as **A**_*m*×*n*_ = **B**_*N*_0_×*n*_^0^ where **b**_*j*_^0^ represents the *j*th column of **B**_*N*_0_×*n*_^0^, which is related to the occurrence of the *i*_*j*_th item in transactions. The support of item *i*_*j*_ can be easily calculated as sup⁡(*X* = *i*_*j*_) = (**b**_*j*_^0^)^*T*^ **b**_*j*_^0^.

Similarly, the support of itemset *X*_*i*,*j*_ = {*i*_*i*_, *i*_*j*_} can be obtained by a simple vector product of the two related vectors because when both *i*_*i*_ and *i*_*j*_ items appear in a given transaction the product of the two related items can be represented by the AND connection of the two items: sup⁡(*X*_*i*,*j*_ = {*i*_*i*_, *i*_*j*_}) = (**b**_*i*_^0^)^*T*^ **b**_*j*_^0^. The main benefit of this approach is that counting and storing the itemsets are not needed; only matrices of the frequent itemsets are generated based on the element-wise products of the vectors corresponding to the previously generated (*k* − 1)-frequent itemsets. Therefore, simple matrix and vector multiplications are used to calculate the support of the potential *k* + 1 itemsets: **S** ^*k*^ = (**B** ^*k*−1^)^*T*^ **B** ^*k*−1^, where the *i*th and *j*th element of the matrix **S** ^*k*^ represent the support of the *X*_*i*,*j*_ = {**L**_*i*_^*k*−1^, **L**_*j*_^*k*−1^} itemset, where **L** ^*k*−1^ represents the set of (*k* − 1)-itemsets. As a consequence, only matrices of the frequent itemsets are generated, by forming the columns of the **B**_*N*_*k*_×*n*_*k*−1__^*k*^ as the element-wise products of the columns of **B**_*N*_*k*−1_×*n*_*k*−1__^*k*−1^; that is, **B**_*N*_*k*_×*n*_*k*−1__^*k*^ = **b**_*i*_^*k*−1^∘**b**_*j*_^*k*−1^, for all *i* ≠ *j*, where *A*∘*B* means the Hadamard product of matrices *A* and *B*.

The concept is simple and easily interpretable and supports compact and effective implementation. The proposed algorithm has a similar philosophy to the Apriori TID [24] method to generate candidate itemsets. None of these methods have to revisit the original data table, **B**_*N*×*n*_^0^, for computing the support of larger itemsets. Instead, our method transforms the table as it goes along with the generation of the *k*-itemsets, **B**_*N*_1_×*n*_1__^1^,…, **B**_*N*_*k*_×*n*_*k*__^*k*^, *N*_*k*_ < *N*_*k*−1_ < ⋯<*N*_1_. **B**_*N*_1_×*n*_1__^1^ represents the data related to the 1-frequent itemsets. This table is generated from **B**_*N*×*n*_^0^, by erasing the columns related to the nonfrequent items, to reduce the size of the matrices and improve the performance of the generation process.

Rows that are not containing any frequent itemsets (the sum of the row is zero) in **B**_*N*_*k*_×*n*_*k*__^*k*^ are also deleted. If a column remains, the index of its original position is written into a matrix that stores only the indices (“pointers”) of the elements of itemsets **L**_*N*_1_×1_^1^. When **L**_*N*_*k*−1_×*k*−1_^*k*−1^ matrices related to the indexes of the (*k* − 1)-itemsets are ordered, it is easy to follow the heuristics of the Apriori algorithm, as only those **L**_*k*−1_ itemsets will be joined whose first *k* − 1 items are identical (the set of these itemsets form the blocks of the **B**_*N*_*k*−1_×*n*_*k*−1__^*k*−1^ matrix).

**Figure 4:**
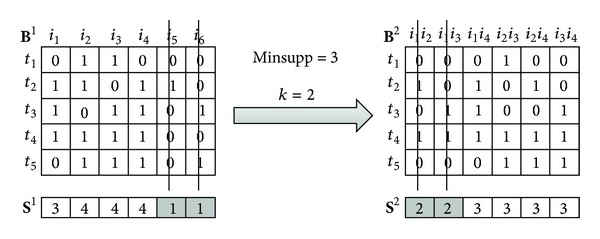
Mining process example using the bit-table representation.

Figure 4 represents the second step of the algorithm, using minsupp = 3 in the *Prune*() procedure.

### 3.2. MATLAB Implementation of the Proposed Algorithm

The proposed algorithm uses matrix operations to identify frequent itemsets and count their support values. Here we provide a simple but powerful implementation of the algorithm using the user friendly MATLAB environment. The MATLAB code 2 (Algorithm 1) and code 3 (Algorithm 2) present working code snippets of frequent closed itemset mining, only within 34 lines of code.

The first code segment presents the second step of the discovery pipeline (see Figure 3). Preprocessed data is stored in the variable *bM* in bit-table format as discussed above. The first and second steps of the iterative procedure are presented in lines 1 and 2, where **S** ^2^ and **B** ^2^ are calculated. The Apriori principle is realized in the *while* loop in lines 4–19. Using the notation in Pseudocode 1, **C** ^**k**^s are generated in lines 10-11 while **L** ^**k**^s are prepared in the loop in lines 12–16.

MATLAB code 3 (Algorithm 2) shows the usually most expensive calculation, the generation of closed frequent itemsets, which is denoted by *extraction of frequent closed itemsets* in Figure 3. Using the set of frequent items as the candidate frequent closed itemsets, our approach calculates the support as the sum of columns (see Section 3.2) and eliminates nonclosed itemsets from the candidate set (line 11). Again, an itemset *J* is a *frequent closed itemset* if it is frequent and there exists no proper superset *J*′⊃*J* such that sup⁡(*J*′) = sup⁡(*J*). This is ensured by the loop in lines 5–9.

## 4. Experimental Results

In this section we compare our proposed method to BiMAX [7], which is a highly recognized reference method within the biclustering research community. As BiMAX is regularly applied to binary gene expression data, it serves as a good reference for the comparison. Using several biological and various synthetic data sets, we show that, while both methods are able to discover all patterns (frequent closed itemsets/biclusters), our pattern discovery approach outperforms BiMAX.

To compare the two mining methods and demonstrate the computational efficiency, we applied them to several real and synthetic data sets. Real data come from various biological studies previously used as reference data in biclustering research [25–28]. For the comparison of the computational efficiency, all biological data sets were binarized. For both the fold-change data (stem cell data sets) and the absolute expression data (Leukemia, Compendium, and Yeast-80) fold-change cutoff 2 is used. Results are shown in Table 1 (synthetic data) and Table 2 (real data), respectively. Both methods were able to discover all closed patterns for all synthetic and real data sets. The results show that our method outperforms BiMAX and provides the best running times in all cases, especially when the number of rows and columns is higher. Biological validation of the discovered patterns together with detailed explanations is given in [28].

## 5. Conclusion

In this paper we have proposed a novel and efficient method to find both frequent closed itemsets and biclusters in high-dimensional binary data. The method is based on a simple bit-table based matrix and vector multiplication approach and ensures that all patterns can be discovered in a fast manner. The proposed algorithm can be successfully applied to various bioinformatics problems dealing with high-density biological data including high-throughput gene expression data.

## Figures and Tables

**Algorithm 1:**
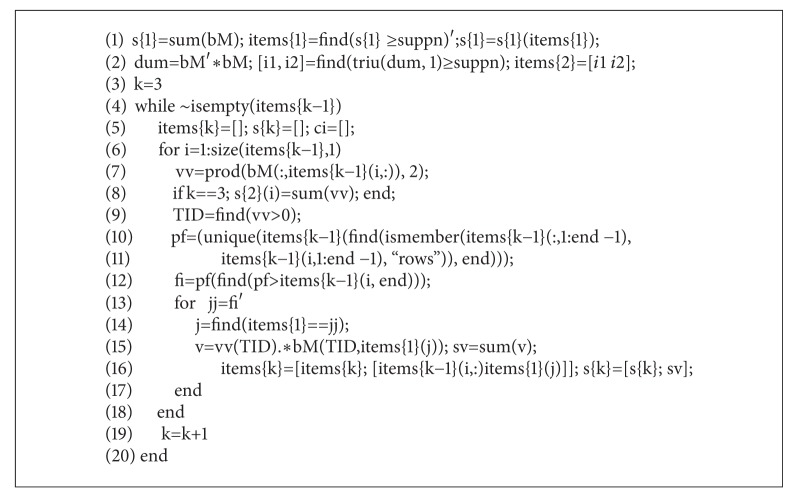
MATLAB code 2: mining frequent itemsets.

**Algorithm 2:**
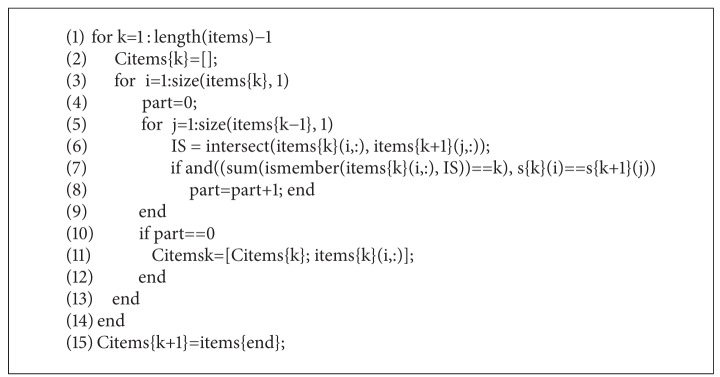
MATLAB code 3: the generation of closed frequent itemsets.

**Table 1:** Performance test using synthetic data.

Size	Density	Minsupp	Number of closed itemsets	Time (s)	Number of BiMAX biclusters	Time (s)
50 × 50	10%	2	78	0.8	78	~1
50 × 50	20%	4	140	1.1	140	~1
50 × 50	50%	15	238	0.9	238	~1
100 × 100	10%	3	337	5	337	~2
100 × 100	20%	7	488	7	488	~2
100 × 100	50%	30	694	9	694	~3
300 × 300	10%	8	437	17	437	~5
300 × 300	20%	22	156	6	156	52
300 × 300	50%	90	1038	40	1038	>600
700 × 700	10%	15	1318	120	1318	195
700 × 700	20%	45	375	33	375	>300
700 × 700	50%	210	283	25	283	>300
1000 × 1000	10%	20	1496	196	1496	>600
1000 × 1000	20%	60	714	92	714	>600
1000 × 1000	50%	290	1030	135	1030	>600

**Table 2:** Test runs using biological data.

Name	Size	Minsupp	Number of closed itemsets	Time (s)	Number of BiMAX biclusters	Time (s)
Compendium	6316 × 300	50	2594	12	2594	~19
StemCell-27	45276 × 27	200	7972	27	7972	~115
Leukemia	125336 × 72	400	3643	147	3643	>600
StemCell-9	1840 × 9	2	177	0.8	177	~1
Yeast-80	6221 × 80	80	3285	8	3285	~17

## References

[1] Agrawal R, Imieliński T, Swami A Mining association rules
between sets of items in large databases.

[2] Göthals B, Zaki MJ Fimi'03: workshop on frequent itemset mining implementations.

[3] Bayardo R, Göthals B, Zaki MJ Fimi'04: workshop on frequent itemset mining implementations.

[4] Madeira SC, Oliveira AL (2004). Biclustering algorithms for biological data analysis: a survey. *IEEE/ACM Transactions on Computational Biology and Bioinformatics*.

[5] Busygin S, Prokopyev O, Pardalos PM (2008). Biclustering in data mining. *Computers & Operations Research*.

[6] Tanay A, Sharan R, Shamir R (2002). Discovering statistically significant biclusters in gene expression data. *Bioinformatics*.

[7] Prelić A, Bleuler S, Zimmermann P (2006). A systematic comparison and evaluation of biclustering methods for gene expression data. *Bioinformatics*.

[8] Tan P-N, Steinbach M, Kumar V (2006). *Introduction to Data Mining*.

[9] Agrawal R, Srikant R Fast algorithms for mining association rules.

[10] Mannila H, Toivonen H, Verkamo AI Efficient algorithms for discovering association rules.

[11] Agrawal R, Mannila H, Srikant R, Toivonen H, Verkamo AI (1996). Fast discovery of association rules. *Advances in Knowledge Discovery and Data Mining*.

[12] Pasquier N, Bastide Y, Taouil R, Lakhal L (1999). Discovering frequent closed itemsets for association rules. *Database Theory—ICDT’99*.

[13] Pei J, Han J, Mao R CLOSET: an efficient algorithm for mining frequent closed itemsets.

[14] Zaki JM, Hsiao C-J CHARM: an efficient algorithm for closed association rule mining.

[15] Grahne G, Zhu J (2003). Efficiently using prefix-trees in mining frequent itemsets. *Proceedings of the Workshop on Frequent Itemset Mining Implementations (FIMI '03)*.

[16] Liu G, Lu H, Lou W, Yu JX On computing, storing and querying frequent patterns.

[17] Wang J, Han J, Pei J CLOSET+: searching for the best strategies for mining frequent closed itemsets.

[18] Vo B, Hong T-P, Le B (2012). DBV-miner: a dynamic bit-vector approach for fast mining frequent closed itemsets. *Expert Systems with Applications*.

[19] Rodriguez-Gonzalez AY, Martinez-Trinidad JF, Carrasco-Ochoa JA, Ruiz-Shulcloper J (2013). Mining frequent patterns and association rules using similarities. *Expert Systems with Applications*.

[20] Han J, Cheng H, Xin D, Yan X (2007). Frequent pattern mining: current status and future directions. *Data Mining and Knowledge Discovery*.

[21] Park JS, Chen MS, Yu PS (1995). *An Effective Hash-Based Algorithm
for Mining Association Rules*.

[22] Amir A, Feldman R, Kashi R (1997). A new and versatile method for association generation. *Information Systems*.

[23] Bayardo RJ Efficiently mining long patterns from databases.

[24] Pach FP, Gyenesei A, Abonyi J (2008). Compact fuzzy association rule-based classifier. *Expert Systems with Applications*.

[25] Gyenesei A, Wagner U, Barkow-Oesterreicher S, Stolte E, Schlapbach R (2007). Mining co-regulated gene profiles for the detection of functional associations in gene expression data. *Bioinformatics*.

[26] Li G, Ma Q, Tang H, Paterson AH, Xu Y (2009). QUBIC: a qualitative biclustering algorithm for analyses of gene expression data. *Nucleic Acids Research*.

[27] Rodriguez-Baena DS, Perez-Pulido AJ, Aguilar-Ruiz JS (2011). A biclustering algorithm for extracting bit-patterns from binary datasets. *Bioinformatics*.

[28] Király A, Abonyi J, Laiho A, Gyenesei A Biclustering of high-throughput gene expression data with bicluster miner.

